# Automated synthesis of radiopharmaceuticals for positron emission tomography: an apparatus for [1-^11^C] labeled carboxylic acid

**DOI:** 10.1155/S1463924695000198

**Published:** 1995

**Authors:** Kazuyoshi Yajima, Hiroyoshi Yamazaki, Hidefumi Kawashima, Sento Ino, Nobuyoshi Hayashi, Yoshihiro Miyake

**Affiliations:** Institute for Biofunctional Research c/o National Cardiovascular Center 7-15-Chome Fujishiro-Dai, Suita City Osaka 565 Japan

## Abstract

A fully automated apparatus for the synthesis and formulation of short-lived ^11^C (t_1/2_ = 20 min)-labeled carboxylic acids for positron emission tomograpy (PET) has been developed. Injectable solutions of [1-^11^C]acetic acid, [1-^11^C]octanoic acid and [1-^11^C]palmitic acid wilh radioactivities of 6.36-8.29 GBq, 0.070-1.43 GBq and 0.42-0.89 GBq were obtained. The preparation time was under 40 min after the end of bombardment. An automatic washing function means that labeled compound of the same or different kinds can be produced several times a day without any maintenance of the system. The control system is sited away from the ‘hot laboratory’, so operator exposure to radiation is minimized.


Journal of Automatic Chemistry, Vol. 17, No. 3 (May–June 1995), pp. 109–114

### Automated synthesis of radiopharmaceuticals for positron emission tomography: an apparatus for [1-$^{11}$C] labeled carboxylic acid

**Kazuyoshi Yajima, Hiroyoshi Yamazaki, Hidefumi Kawashima, Sento Ino, Nobuyoshi Hayashi and Yoshihiro Miyake**

*Institute for Biofunctional Research, c/o National Cardiovascular Center, 7-15-Chome, Fujishiro-Dai, Suita City, Osaka 565, Japan*

*A fully automated apparatus for the synthesis and formulation of short-lived $^{11}C$ ($t_{1/2}$ = 20 min)-labeled carboxylic acids for positron emission tomography (PET) has been developed. Injectable solutions of [1-$^{11}$C]acetic acid, [1-$^{11}$C]octanoic acid and [1-$^{11}$C]palmitic acid with radioactivities of 6·36–8·29 GBq, 0·070–1·43 GBq and 0·42–0·89 GBq were obtained. The preparation time was under 40 min after the end of bombardment. An automatic washing function means that labeled compounds of the same or different kinds can be produced several times a day without any maintenance of the system. The control system is sited away from the 'hot laboratory', so operator exposure to radiation is minimized.*

### Introduction

Positron emission tomography (PET) is a non-invasive diagnostic imaging technique in which short-lived radioactive tracers are used to measure the anatomical distribution and kinetics of *in vivo* biochemical processes [1]. [1-$^{11}$C]Labeled carboxylic acids are useful radiotracers in PET studies; [1-$^{11}$C]labeled acetate and palmitate have been used extensively as tracers for monitoring cardiovascular functions [2–4]. Although a number of semi- or fully-automated systems for the production of $^{11}$C-labeled carboxylic acids have been developed [5–11], they have drawbacks in that they need manual washing before re-use and/or require the attendance of specialist operators. Thus, a reliable system, which would reproducibly deliver radiopharmaceuticals on a routine and repetitive basis, is required.

The design and construction of a fully automated apparatus for producing [1-$^{11}$C]labeled carboxylic acids is described in this paper. The apparatus has been applied to the repetitive production of [1-$^{11}$C]acetic acid, [1-$^{11}$C]octanoic acid and [1-$^{11}$C]palmitic acid.

### Automated synthesis apparatus of [1-$^{11}$C]labeled carboxylic acid

### *Hardware*

The automated apparatus consists of a synthesis system and a control system. The synthesis system, an auto-manual switch box, and I/O boards are placed in a radiation shielded room (hot laboratory). The computer and its peripherals are in a separate room. The synthesis system consists of a series of units which supply reagents, perform reactions, separate and purify $^{11}$C-labeled carboxylic acid, and formulate an injectable solution of the $^{11}$C-labeled carboxylic acid. The operation of these units is performed either automatically by the control system or manually through the auto-manual switch box (manual operation is useful for cold experiments and maintenance). As the solenoid valves and other devices, for example the reagent supply unit and reaction unit, are set on a punched metal board, modification and maintenance of the hardware is simple.

### *Synthesis apparatus*

A diagram of the apparatus is shown in figure 1.

### *Reagent supply units*

The reagent supply unit has seven reservoirs (1–7 in figure 1) for reagent solutions and solvents. Each solution or solvent in the reservoirs is stored under argon atmosphere, and can be transferred to the flasks (8–11 in figure 1) in one or two steps. In the latter case, the liquid is allowed to flow from the reservoir into a volumetric tube by argon gas pressure. When a photosensor (31–37 in figure 1) on the tube detects the liquid, the valve at the lower end of the reservoir is switched to stop the flow of the liquid. The contents of the tube are transferred into the reaction flask by argon gas pressure. By the use of this volumetric device, the same volume of liquid may be repeatedly measured out and added to the flask; even moisture- or air-sensitive liquids can be handled. After a synthetic run, all of the main flow lines and the flasks (8, 9 and 13 in figure 1) can be washed by passing a washing solvent stored in reservoir 4, and then dried with dry nitrogen. In this way radiopharmaceuticals of the same or different kind can be produced during a day without interruption due to maintenance.

### *Reaction unit*

The reaction unit has two flasks (8 and 9 in figure 1). Flask 1 (8 in figure 1) carries out the Grignard coupling, and is placed in a dry box (45 in figure 1) to keep off any moisture. Flask 2 (9 in figure 1) quenches the reaction and evaporates the solvent. Both flasks are about 2 ml in volume and have jackets through which the heating/cooling fluid is circulated under temperature control by a thermostat (23 in figure 1). The mixing of the reaction mixture in flask 1 is accomplished by bubbling

0142-0453/95 $10.00 © 1995 Taylor & Francis Ltd.

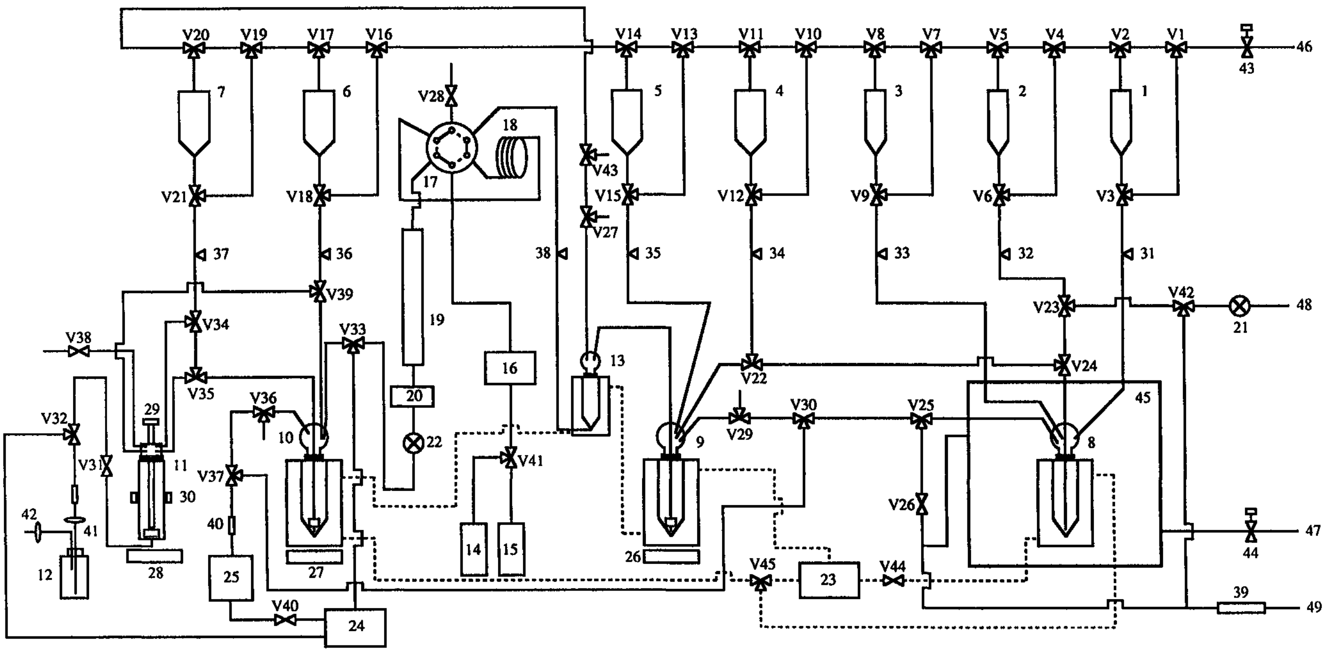

*Figure 1. Diagram of the synthesis system: V1 $\sim$ V45 = solenoid valve 1 $\sim$ 45; 1 $\sim$ 7 = reservoir 1 $\sim$ 7; 8 $\sim$ 11 = flask 1 $\sim$ 4; 12 = vial; 13 = degas flask; 14 and 15 = tank 1 $\sim$ 2 of HPLC; 16 = HPLC pump; 17 = rotary six-way valve; 18 = sample loop; 19 = HPLC column; 20 = UV detector; 21 and 22 = radiation detector 1 and 2; 23 = thermostat; 24 = drainage tank; 25 = vacuum pump; 26 $\sim$ 28 = magnetic stirrer; 29 = pH sensor; 30 = level sensor; 31 $\sim$ 38 = photosensor 1 $\sim$ 8; 39 and 40 = sodalime 1 and 2; 41 and 42 = filter 1 and 2; 43 and 44 = mass flow controller 1 and 2; 45 = dry box; 46 and 47 = argon gas line; 48 = $^{11}CO_2$ gas line; 49 = waste gas line.*

argon gas. The bubbling rate is controlled with a mass flow controller (43 in figure 1). The mixing of the reaction mixture in flask 2 is accomplished by a magnetic stirrer (26 in figure 1), and the evaporation in flask 2 is carried out under vacuum created by the vacuum device (25 and 40 in figure 1). Transfer of the reaction mixture in flask 1 to flask 2, or the solution in flask 2 to the purification unit, is carried out by the use of argon gas pressure.

### *Purification unit*

The purification unit is composed of two systems: a degassor system which also serves as a part of an injecting system into the high performance liquid chromatography (HPLC) column and an HPLC system. The degassor system consists of a degassing flask (13 in figure 1; about 2 ml in volume), a photosensor (38 in figure 1), a rotary six-way valve (17 in figure 1) and a sample loop (18 in figure 1). The degassing flask has a jacket through which a heating/cooling fluid is circulated. The HPLC system consists of a pump (16 in figure 1), an HPLC column (19 in figure 1), a UV detector (20 in figure 1) and a radiation detector (22 in figure 1). The concentrate obtained in flask 2 is transferred to the degassing flask, degassed and then sent to the sample loop of an HPLC apparatus, through the photosensor (38) and a rotary six-way valve. When the photosensor (38) detects the end of the solution, the rotary six-way valve rotates its position automatically and the mixture in the sample loop is then loaded to the HPLC column by the HPLC pump (16). The fraction which contains the product is detected by the radiation detector (22) and collected in a flask (10 in figure 1).

### *Pharmacological formulation unit*

The pharmacological formulation unit has two flasks (10 and 11 in figure 1) and one vial. Flask 3 (10 in figure 1) has a jacket which is thermostated by circulating the heating/cooling fluid through it. The contents of flask 3 are concentrated under reduced pressure stirred with a magnetic stirrer (27 in figure 1) and heated in order to remove any organic solvent. The residual solution in flask 3 is transferred to flask 4 (11 in figure 1), which is equipped with a magnetic stirrer (28 in figure 1), a pH sensor (29 in figure 1) and a level sensor (30 in figure 1). The pH and volume of the solution can be adjusted in flask 4 to the desired values by adding an alkaline solution, an albumin solution or saline. The resultant solution is filtered through a 0.22 µm membrane filter (41 in figure 1) into a product vial (12 in figure 1) to give a solution of a $^{11}$C-labeled compound in a ready-to-use form for a PET study after an appropriate quality assurance procedure.

### *Control system*

### *Computer and interface hardware*

The apparatus is controlled with a personal computer (PC-9821Ap, NEC); an adaptor (RS422) in the computer controls I/O boards, Optomux (Opto22, USA). Six Optomux digital and two analogue brain boards, each with 16 I/O channels, are used. Each board has a unique address to help identify every device in the system, for example five digital output boards give 80 switches for operating valves and relays. One digital input board reads the status of 16 indicator lamps that are used for monitoring photosensors, the level sensor, and the position of the rotary six-way valve. The analogue boards are for reading status information from the system, including radioactivity, pH, UV absorbance, the rates of the mass flow controllers and pressure, and for writing information to set rates of the mass flow controllers.

### *Software*

The software was developed with a development program called '*Hyakuninriki*' (Asahi Electronics Co. Ltd, Japan), which operates under MS-DOS. The software has functions for control logic and graphic displays. The program consists of a series of connected control blocks with the flow depending on the control logic sequence. More than 140 control blocks of 16 different types are used and each block is programmed to perform a function,

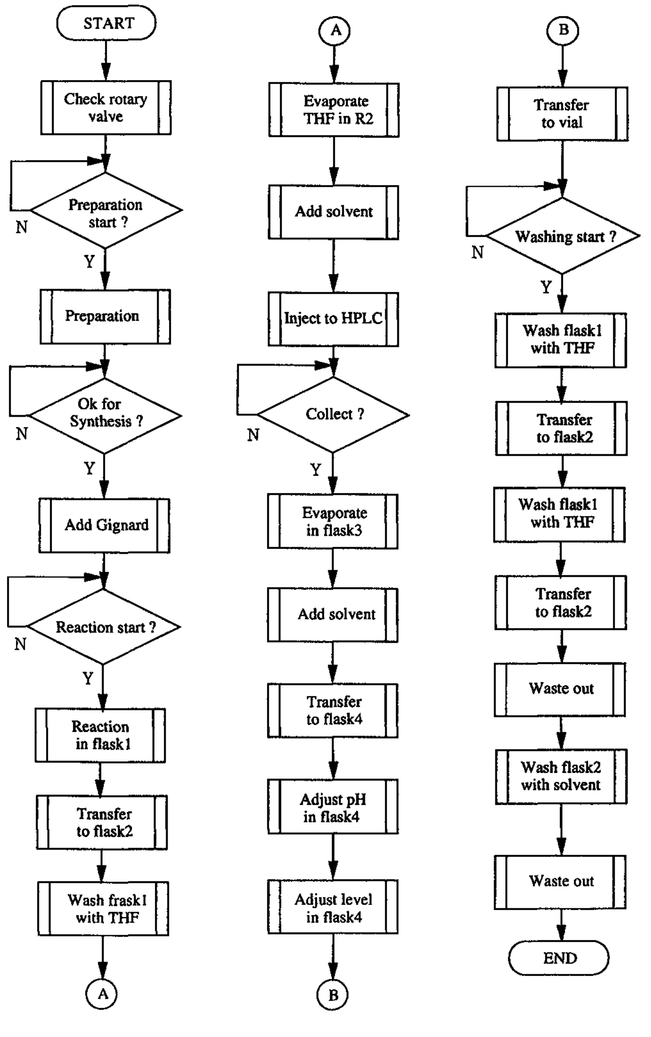

*Figure 2. Flowchart of the total operation: The preparation of the apparatus process contains subroutines of 'Check rotary valve' and 'Preparation'. The Grignard reaction process contains subroutines from 'Add Grignard' to 'Add solvent'. The purification process contains subroutines from 'Inject to HPLC' to 'Transfer to flask 4'. The formulation process contains subroutines of 'Adjust pH in flask 4', 'Adjust level in flask 4' and 'Transfer to vial' and the washing process contains subroutines from 'Wash flask 1 with THF' to 'Waste out'.*

such as controlling a switching sequence or reading the status of sensors. The program consists of five processes, for example preparation of the apparatus, Grignard reaction, purification, formulation and washing processes (see figure 2). The preparation of the apparatus process is controlled by the method of close loop and a time sequential method. The Grignard reaction process and the purification process are performed by sequential control using a signal from the photosensor and a time sequential method. The formulation process is controlled by the method of close loop, and the washing process is controlled by the method of close loop, sequential control using the signal of photosensors and a time sequential method.

### Operating procedure

### *Layout of the hot experiment zone*

The layout of the hot-experiment zone is shown in figure 3; the four rooms used (cyclotron room, hot laboratory, pass room and control room) are isolated from each other. The values of the radioisotope calibrators can be monitored with two video cameras in the control room.

### *Preparation for synthesis*

The reservoirs (1–7 in figure 1) of the apparatus are filled as follows: reservoir 1–3 (0·2M solution of Grignard reagents in anhydrous THF), reservoir 4 (anhydrous THF), reservoir 5 (a mixture of 1N HCl and the eluent for HPLC = 1:1(v/v)), reservoir 6 (7% $NaHCO_3$ solution in the case of acetic and octanoic acid or an albumin solution in the case of palmitic acid), reservoir 7 (saline). The HPLC system is turned on. The temperature of the fluid in the jacket is set and the position of the rotary six-way valve is set to the correct starting position. Argon gas flow rates of the mass flow controllers 1 and 2 are set to 50 ml/min. The degassing system is purged with argon gas (flow line: 46-43-V43-V27-13-38-17-18-V28 in figure 1) for 15 min, and then flask 1 is purged with argon gas (flow line: 46-43-V1-V3-31-8-V25-V26-39-49 in figure 1) by switching V1 and V3.

### *Reaction of $^{11}CO_2$ with Grignard reagents*

Production of $^{11}CO_2$ is accomplished via $^{14}N(p, \alpha)^{11}C$ reaction by proton bombardment (18 MeV, 15 μA) on $N_2$ gas target (14.7 kg/cm$^2$) using a cyclotron-target system (CYPRIS HM-18, Sumitomo Heavy Industries Co. Ltd, in figure 2). After irradiation, the $^{11}CO_2$-containing target gas is transferred to a $^{11}CO_2$ trap (AMCT 01, NKK Corp. in figure 2). [$^{11}$C] Carbon dioxide is trapped in a coiled tube dipped into liquid argon. Then, the trapped $^{11}CO_2$ is transferred to the synthesis apparatus with heating the coil and by the flow of He gas (20 ml/min). The radioisotope detector on the inlet line (CsI scintillator (gas in) in figure 2) is used to monitor the release of radioactivity from the trap. The outlet of reservoir (1, 2 or 3 in figure 1) is opened by switching valves (V2 and V3, V5 and V6, or V8 and V9 in figure 1) to measure the Grignard solution. And the measured solution is transferred into flask 1. This

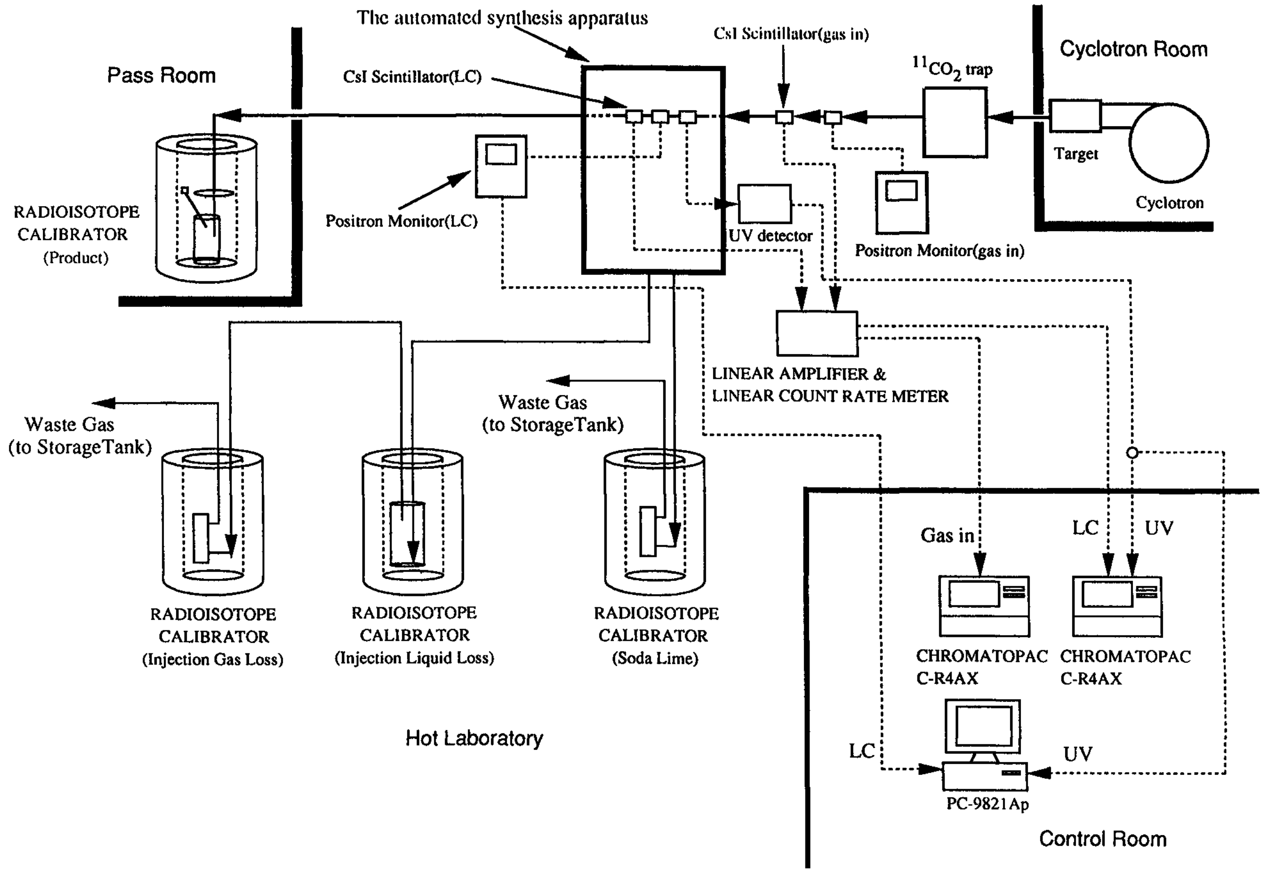

*Figure 3. Layout for hot experiments.*

operation is repeated three times immediately after the release of radioactivity. Valves V42, V23, V25 and V26 are then switched to direct the flow of $^{11}CO_2$ gas to flask 1. The $^{11}CO_2$ gas is bubbled through the Grignard solution for 2 min 30 sec; a sodalime trap (39 in figure 1) prevents any radioactive gas from leakage. Then, the reaction mixture in flask 1 is transferred to flask 2 by switching valves V42, V23, V25, V26, V1, V3, V24 and V29 (flow line: 46-43-V1-V3-8-V24-V22-9-V29). Flask 1 is rinsed once with 1·0 ml of THF from reservoir 4 and the washings are also transferred to flask 2.

*Quenching and evaporation*

The power switches of the vacuum pump and the magnetic stirrer (26 in figure 1) are turned on. Then valves V30 and V40 are switched, and the reaction mixture is concentrated under reduced pressure. After evaporation, the vacuum pump is stopped, and in order to break the vacuum, valve V29 is switched. The residue in flask 2 is dissolved in 2·0 ml of a solution from reservoir 5 in a similar manner (flow line: 46-43-5-V15-35-9-V29) as above.

*Purification and formulation*

The product of the previous step is purified and separated via the degassor system and HPLC system as follows:

(1) Valves V13, V15 and V27 are switched to deliver the solution in flask 2, which may contain air bubbles, to the degassing flask with argon gas pressure (flow line: 46-43-V13-V15-35-9-13-V27).
(2) Valve V27 is switched, and the degassed solution is sent to the sample loop via photosensor (38 in figure 1) and the rotary six-way valve.
(3) When the end of the solution is detected by photosensor (38 in figure 1), the rotary six-way valve is switched and the solution in the sample loop is loaded to the HPLC column.
(4) The eluate from the HPLC is monitored by the UV and radiation detectors on the HPLC-eluate line and the fraction containing [1-$^{11}$C]carboxylic acid is collected in flask 3 by switching valves V33 and V36. A magnetic stirrer (27 in figure 1) is then started.

After the collection is finished, evaporation of the contents of flask 3 is performed by switching valves V37, V40 and the vacuum pump. When the evaporation is completed, the vacuum pump is stopped, and valve V36 is switched in order to release the vacuum. The residue in flask 3 is dissolved in $NaHCO_3$ solution (2·0 ml) transferred from reservoir 6 (flow line: 46-43-V17-6-V18-V39-10), and then the solution in flask 3 is transferred to flask 4 by switching valves V16, V18, V39 and V28 (flow line: 46-43-V16-V18-36-V39-10-V35-11-V38).

The magnetic stirrer 3 is switched on and the pH value of the solution in flask 4 is measured with the pH sensor. The pH value is adjusted, if necessary, to the range from 8·5 to 9·0 by the addition of the $NaHCO_3$ solution from reservoir 6 and the resulting solution in flask 4 is then diluted with saline from reservoir 7 by adding it to the solution until the level sensor, which is set beforehand to a volume of 10 ml, is activated. Finally the product is filtered through a 0·22 μm sterile filter by applying argon gas pressure (flow line: 46-43-V19-V21-V34-V35-V11-41-12) into a sealed vial placed in a radioisotope calibrator measuring the radioactivity of the final product in passroom (figure 2). The product is then analysed by an analytical HPLC to confirm the quality.

*Table 2. Radioactivities of the successive production of [1-$^{11}$C]octanoic acid.*

| Run # | *Radioactivity (GBq) |
|---|---|
| 1 | 1·22 |
| 2 | 0·70 |

*At the end of formulation.

### *Washing of the apparatus*

Washing of the apparatus can be started immediately after the final solution of the product has been obtained. The position of the rotary six-way valve is set to the starting position. The washing of flask 1 is performed by sending 2·0 ml of anhydrous THF from reservoir 4 and the washings are transferred to flask 2 in a similar manner as above. These operations are repeated twice. The washing solution in flask 2 is evaporated to dryness under reduced pressure in a similar manner mentioned above. After evaporation, flask 2 is washed with 2·0 ml of a solution from reservoir 5. These operations are repeated twice. Finally the washings are transferred to a waste tank out of the apparatus by argon gas pressure (flow line: 46-43-V13-V15-35-9-13-38-17-V28).

### Results and discussion

With the apparatus described, injectable solutions of [1-$^{11}$C]labeled carboxylic acids with different chain lengths, e.g. [1-$^{11}$C]acetic acid, [1-$^{11}$C]octanoic acid and [1-$^{11}$C]palmitic acid, were obtained. Table 1 shows the ranges of the radioactivities and radiochemical purity of [1-$^{11}$C]labeled carboxylic acids produced on several runs. The total synthesis time from the end of bombardment to the end of formulation was within 40 min. $^{11}CO_2$ was produced via $^{14}N(p, \alpha)^{11}C$ reaction by proton bombardment (18 MeV, 15 μA) on a 14·7 kg/cm$^2$ $N_2$ gas target for 40 min.

The apparatus washes automatically, so labeled compounds of the same or different kinds can be produced several times during a day without maintenance of the system. Table 2 shows the result of repetitive synthesis of [1-$^{11}$C]octanoic acid. [1-$^{11}$C]octanoic acid was obtained twice a day without maintenance of the apparatus.

Although [1-$^{11}$C]octanoic acid has been selected and synthesized repetitively to show the performance of the apparatus, the apparatus can be used for repetitive synthesis of other [1-$^{11}$C]labeled carboxylic acids with only minor changes on the software and hardware. Finally the operator's exposure to radiation is minimized.

*Table 1. Radioactivities of the [1-$^{11}$C] labeled carboxylic acids.*

| Compounds | *Radioactivity (GBq) | Radiochemical purity (%) |
|---|---|---|
| [1-$^{11}$C]acetic acid | 6·36–8·29 | >99 |
| [1-$^{11}$C]octanoic acid | 0·70–1·43 | >99 |
| [1-$^{11}$C]palmitic acid | 0·42–0·89 | >99 |

*At the end of formulation.

### Experimental

### *Materials and reagents*

As Grignard reagents are very moisture sensitive, they were injected into reservoir(s) 1, 2 and/or 3, capped with a Teflon-lined septum and flushed with argon, before the start of synthesis. Tetrahydrofuran (THF) was freshly distilled over lithium aluminium hydride under argon atmosphere just before use.

### *Production of [1-$^{11}$C]acetic acid*

The reservoirs of the apparatus were filled as follows (the quantities in parentheses are those used for one synthesis)—reservoir 1: 0·2 M $CH_3MgBr$/THF (0·5 ml), reservoir 4: THF (1·0 ml), reservoir 5: a mixture of 1N HCl and 0.025 M NaCl (1:1 (v/v)) (0·5 ml), reservoir 6: 7% $NaHCO_3$aq. (2·0 ml), reservoir 7: saline (5·0 ml). The fluid in the thermostat (23 in figure 1) was cooled to the temperature of −26°C, and valves V44 and V45 were switched in order to cool flask 1 only. The HPLC conditions used were for preparation, column: Waters protein pack G-QA 20 I.D.*100 mm, eluent: 0·025M NaCl, flow rate: 5·0 ml/min, detector (UV): 214 nm, temperature: room temperature, retention time: *c.* 11 min and radiodetector: Aloka Positron Monitor TCS-R81-3454 and for analysis, column: YMC-Pack ODS-AQ, AQ-303 4·6 I.D.*250 mm, eluent: 20 mM $H_3PO_4$–$NaH_2PO_4$ (pH2·8), flow rate: 0·7 ml/min, detector (UV): 214 nm, temperature: room temperature, retention time: *c.* 8·5 min, and radiodetector: Aloka Gamma Detector FGD-102 and Radio Analyser RLC-700.

### *Production of [1-$^{11}$C]octanoic acid*

The reservoirs of the apparatus were filled as follows, (the quantities in parentheses are those used in one synthesis)–reservoir 2: 0·2M $C_7H_{15}MgBr$/THF (0·5 ml), reservoir 4: THF (1·0 ml), reservoir 5: a mixture of 1N HCl and $CH_3CN$:$H_2O$:6N HCl = 1:1:0·002 (v/v) (0·5 ml), reservoir 6: 7% $NaHCO_3$aq. (2·0 ml), reservoir 7: saline (5·0 ml). The reaction was carried out at room temperature. The HPLC conditions used were, for preparation, column: YMC-ODS-AQ 10 I.D.*250 mm, eluent: $CH_3CN:H_2O:6$ N HCl = 1:1:0·002 (v/v), flow rate: 5·0 ml/min, detector (UV): 214 nm, temperature room temperature, retention time: *c.* 11 min, and radiodetector: Aloka Positron Monitor TCS-R81-3454 and, for analysis, column: YMC-ODS-AQ 4·6 I.D.*250 mm, eluent: $CH_3CN:H_2O:6$N HCl = 600:400:1 (v/v), flow rate: 1·0 ml/min, detector (UV): 214 nm, temperature: room temperature, retention time: *c.* 12 min, and radiodetector: Aloka Gamma Detector FGD-102 and Radio Analyser RLC-700.

*Production of [1-$^{11}$C]palmitic acid*

The reservoirs of the apparatus were filled as follows, (the quantities in parentheses are those used in one synthesis)–reservoir 3: 0·2 M $C_{15}H_{31}MgBr$/THF (0·5 ml), reservoir 4: THF (1·0 ml), reservoir 5: a mixture of 1N HCl and $CH_3CN:H_2O$ = 95:7 (v/v) (0·5 ml), reservoir 6: Albumin solution (2·0 ml), reservoir 7: saline (5·0 ml). The fluid in the thermostat (23 in figure 1) was warmed to the temperature of 56°C, and the outlet of it was opened by switching valve V45. Flask 1, 2 and the degas flask were warmed to the same temperature. The HPLC conditions used were, for preparation, column: YMC-ODS-AQ 10 I.D.*250 mm, eluent: $CH_3CN:H_2O$ = 95:7 (v/v), flow rate: 5·0 ml/min, detector(UV): 214 nm, temperature: room temperature, retention time: *c.* 12 min, and radiodetector: Aloka POSITRON MONITOR TCS-R81-3454 and, for analysis, column: YMC-ODS-AQ 10 I.D.*250 mm, eluent: $CH_3CN:H_2O$ = 95:7 (v/v), flow rate: 0·8 ml/min, detector (UV): 214 nm, temperature: room temperature, retention time: *c.* 12 min, and radiodetector: Aloka Gamma Detector FGD-102 and Radio Analyser RLC-700.

*Successive production of [1-$^{11}$C]octanoic acids*

All the conditions are the same as above for the production of [1-$^{11}$C]octanoic acid. The apparatus is washed between sequential production of[1-$^{11}$C]octanoic acids.

### Acknowledgments

This work was partly supported by a grant from the Japan Health Science Foundation. The authors wish to acknowledge Dr Teruo Omae, President of the National Cardiovascular Center, for supporting this work and to express their thanks to Dr N. Hashimoto for reviewing the manuscript and Dr D. G. Cork of Takeda Chemical Industries Ltd for useful discussions. The operation of the cyclotron by Mr N. Ejima is also acknowledged and appreciated.

### References

1. Phelps, M. E., Mazziotta, J. C. and Schelbert, H. R., *Positron Emission Tomography and Autoradiography* (Raven Press, New York, 1986).
2. Buck, A., Wolpers, H. G., Hutchins, G. D., Savas, V., Mangner, T. J., Nguyen, N. and Schwaiger, M., *Journal of Nuclear Medicine*, **32** (1991), 1950.
3. Ng, C. K., Huang, S-C., Schelbert, H. R. and Buxton, D. B., *American Journal of Physiology*, **266** (1994), H1304.
4. Buckman, O. B., VanBrocklin, H. F., Dence, C. S., Bergman, S. R., Welch, M. J. and Katzenellenbogen, J. A., *Journal of Nuclear Medicine*, **37** (1994), 2481.
5. Welch, M. J., Dence, C. S., Marshall, D. R. and Kilbourn, M. R., *Journal of Labelled Compounds and Radiopharmaceuticals*, **20** (1983), 1087.
6. Victor, W., Pike, L. H., Cyril, B. and John, C. C., *International Journal of Applied Radiation and Isotopes*, **35** (1984), 623.
7. Del Fiore, G., Peters, J. M., Quaglia, L., Piette, J. L., Cantineau, R., De Landsheere, C., Raets, D. and Rigo, P., *Journal of Radioanalytical Chemistry*, **87** (1984), 1.
8. Douglas, M. J., Richard, L. E. and Siya, R., *International Journal of Applied Radiation and Isotopes*, **36** (1985), 672.
9. Takahashi, T., Ido, T., Iwata, R., Hatano, K., Nakanishi, H., Shinohara, M. and Iida, S., *Applied Radiaton and Isotopes*, **39** (1988), 659.
10. Norenberg, J. P., Simpson, N. R., Dunn, B. B. and Kiesewetter, D. O., *Applied Radiation and Isotopes*, **43** (1992), 943.
11. Isiwata, K., Ishii, S., Sasaki, T., Senda, M. and Nozaki, T., *Applied Radiation and Isotopes*, **44** (1993). 761.

